# Use of methylene blue discography in transforaminal endoscopic discectomy for cranially migrated lumbar disc herniation: A case report

**DOI:** 10.1016/j.radcr.2026.08.035

**Published:** 2026-09-04

**Authors:** Marcus Vinicius Brito Marques, Fabrício Guedes Machado, Jorge Luis Marques Ponte, Roney Souza de Sant’Anna, Marcelo Botelho Soares de Brito

**Affiliations:** a Department of Orthopedics and Traumatology, MaterDei Hospital, Salvador, Bahia, Brazil; b Neuromusculoskeletal System Unit, Professor Edgard Santos University Hospital, Salvador, Bahia, Brazil; c Department of Neurosurgery, MaterDei Hospital, Salvador, Bahia, Brazil; d Department of Spine Surgery, Articulare Clinic, Belém, Pará, Brazil

**Keywords:** Intervertebral disc displacement, Methylene blue, Discography, Endoscopic spine surgery, Transforaminal discectomy, Intraoperative neurophysiological monitoring

## Abstract

Cranially migrated foraminal lumbar disc herniations remain technically demanding during transforaminal endoscopic discectomy, because the narrow working corridor restricts direct visualization of extruded fragments. We describe a 61-year-old woman with refractory left-sided radiculopathy and progressive motor deficit. Magnetic resonance imaging showed a left L4-L5 foraminal disc extrusion with an annular tear and cranial migration to the upper segment of the neuroforamen, classified as Lee zone 1, causing severe foraminal narrowing with compression and deformity of the exiting nerve root. Before resection, intradiscal injection of iodinated contrast and methylene blue under fluoroscopic guidance stained 2 extruded fragments and allowed targeted removal without neural injury. Multimodal intraoperative neurophysiological monitoring documented progressive recovery of motor-evoked potential amplitudes during decompression. The patient was discharged the same day. At 1 month the Oswestry Disability Index had improved from 86% to 10% and the visual analog scale from 10 to less than 1, and the improvement was sustained at 8 months. In this patient, methylene blue discography appeared to facilitate localization of the migrated fragments within a constrained foraminal corridor, while neurophysiological monitoring added real-time functional assessment of neural decompression. Comparative studies are needed before either adjunct can be recommended routinely in this herniation subtype.

## Introduction

Lumbar disc herniation is among the most common causes of radiculopathy and functional disability in adults, with the lower lumbar levels disproportionately affected [1]. Foraminal and extraforaminal herniations account for a smaller yet clinically distinct subset, compressing the exiting nerve root and dorsal root ganglion rather than the traversing root and often producing more severe radicular pain and earlier motor deficits compared with central or paracentral herniations [2].

Transforaminal endoscopic discectomy has gained acceptance as a minimally invasive alternative for lumbar disc surgery, offering effective decompression with limited soft-tissue disruption and shorter hospitalization [3]. Cranially migrated foraminal herniations, however, present a specific technical problem. The working channel trajectory and the limited foraminal corridor restrict direct visualization of the fragment, increasing the risk of incomplete resection and, consequently, of symptom persistence or early recurrence [4,5]. Foundational descriptions of transforaminal endoscopic technique by Yeung and Tsou incorporated intradiscal dye injection as part of the initial discographic step, establishing a methodological basis for chromatic discrimination of disc tissue during endoscopic access [6]. Subsequently, selective endoscopic intervertebral nuclectomy using indigo carmine was described by Kim et al. [7], and methylene blue has since been applied in transforaminal endoscopic procedures with reported advantages in fragment delineation [8,9]. Methylene blue penetrates disc tissue that comes into contact with the injectate and produces a blue coloration that contrasts with surrounding epidural fat and neural structures, so that disc material can be distinguished from non-disc structures in the endoscopic field [9].

We report a case in which methylene blue discography was used as an intraoperative adjunct during transforaminal endoscopic discectomy for a cranially migrated L4-L5 foraminal disc extrusion. The procedure was performed under continuous intraoperative neurophysiological monitoring (IONM), integrating dye-guided anatomical orientation with real-time functional feedback in a technically demanding foraminal corridor. To our knowledge, this anatomical subtype has rarely been addressed in prior dye-assisted endoscopy reports, in which most descriptions involve central or paracentral herniations approached through standard corridors. In foraminal migration, the working axis runs nearly parallel to the displaced fragment, making chromatic discrimination proportionally more valuable.

## Case presentation

This case report was approved by the local institutional ethics committee, and written informed consent for publication was obtained from the patient. The report was prepared in accordance with the CARE guidelines.

**Fig. 1:**
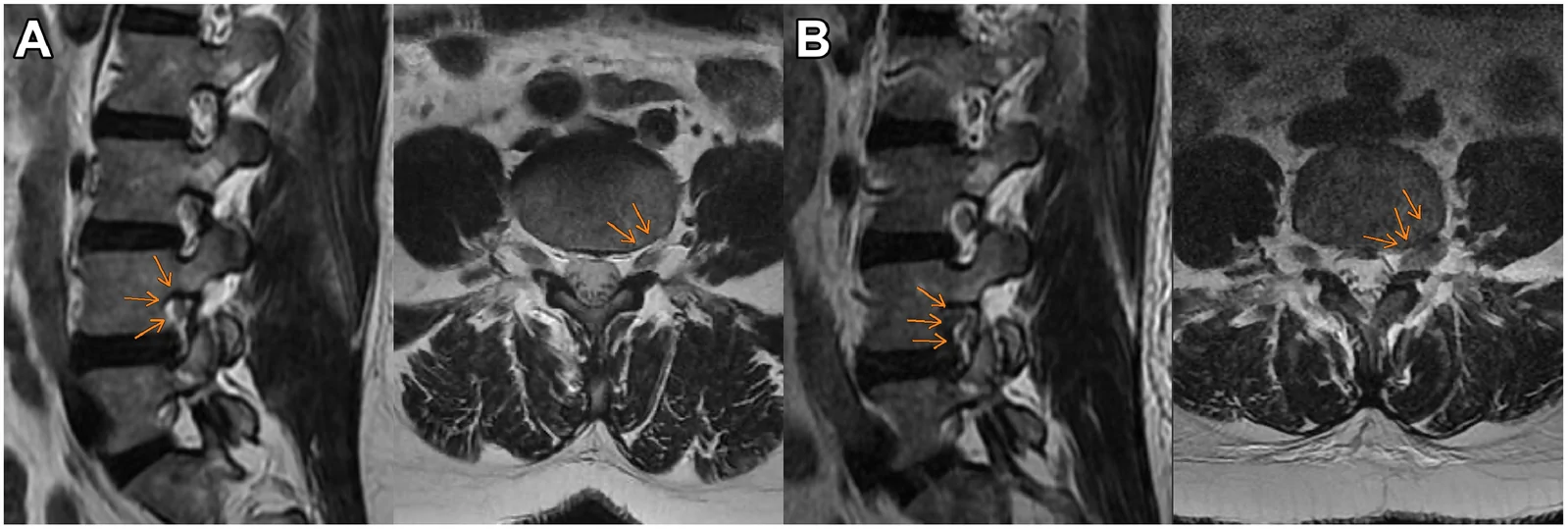
Lumbar magnetic resonance imaging demonstrating progression of L4-L5 disc pathology. (A) Initial study, sagittal and axial T2-weighted images, showing diffuse disc bulging at L4-L5 without significant neural compression (arrows). (B) Repeat study obtained 3 months later, sagittal and axial T2-weighted images at the same level, showing a left foraminal disc extrusion with cranial migration of the fragment into the upper segment of the neuroforamen (arrows), severe foraminal narrowing, and compression with deformity of the exiting left nerve root.

A 61-year-old woman presented with low back pain radiating to the left lower limb and progressive functional limitation refractory to conservative treatment. Pain intensity was measured at 6 out of 10 on the visual analog scale (VAS) [10], with an Oswestry Disability Index (ODI) [11] of 42%. Initial lumbar Magnetic Resonance Imaging (MRI) demonstrated multilevel degenerative changes compatible with spondylodiscarthrosis and mild disc bulging at L4-L5, without evidence of significant neural compromise at that time (Fig. 1A). Pharmacological analgesia and physiotherapy were continued while a lumbar infiltration was scheduled.

Three months later, the patient reported sudden and marked worsening of radicular symptoms following minor domestic exertion, with VAS increasing to 10 out of 10 and ODI rising to 86%, resulting in complete inability to ambulate. Pregabalin 300 mg/day and tramadol 400 mg/day provided inadequate relief. Clinical examination revealed an antalgic gait requiring a walking aid, restricted lumbar range of motion, and use of a lumbosacral orthosis. Neurological examination revealed paresthesia over the posterior aspect of the left thigh, allodynia along the medial and anterior aspects of the left leg, a positive left straight-leg raise test, and motor weakness consistent with involvement of the left L4 and L5 nerve roots, with left knee extension, ankle dorsiflexion, and extensor hallucis longus strength graded as Medical Research Council (MRC) grade 3/5. Repeat lumbar MRI, including sagittal and axial T2-weighted sequences, demonstrated an extruded disc herniation at L4-L5 that had migrated cranially to reach the upper segment of the neuroforamen, associated with a tear of the annulus fibrosus. The extruded material showed signal characteristics consistent with disc material and clear continuity with the parent disc. It caused severe narrowing of the left neural foramen, with compression and deformity of the exiting nerve root, and adjacent inflammatory changes were present (Fig. 1B).

**Fig. 2:**
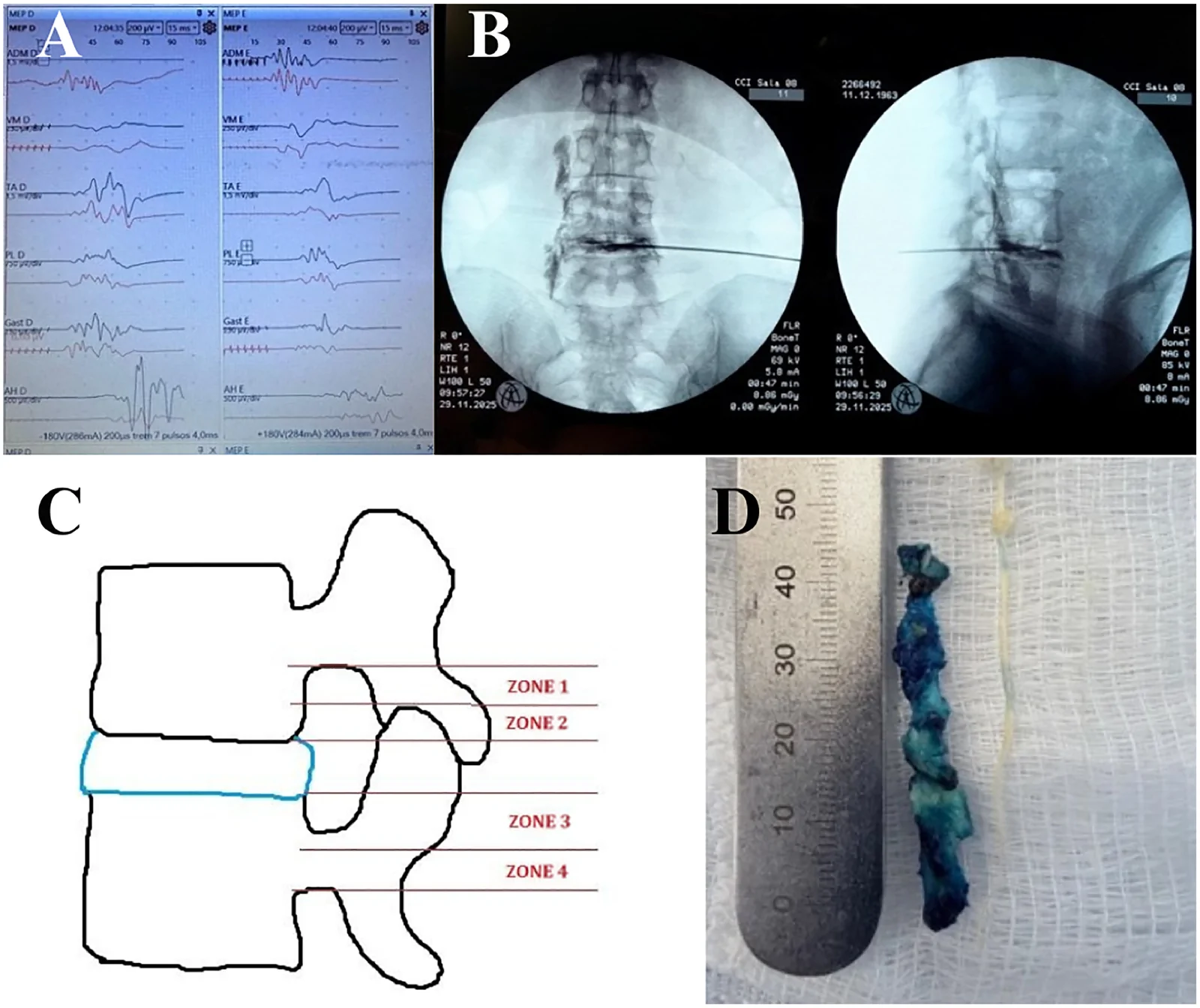
Intraoperative findings and anatomical correlates during transforaminal endoscopic discectomy. (A) Multimodal intraoperative neurophysiological monitoring before decompression, showing reduced amplitudes of left-sided motor-evoked potentials, predominantly in L4-innervated musculature, with stable contralateral recordings. (B) Anteroposterior and lateral fluoroscopic images confirming needle position within the L4-L5 disc space for discography with iodinated contrast and methylene blue. (C) Schematic of the Lee classification of disc migration, with zone 1 corresponding to the present case. (D) Extruded disc fragments stained with methylene blue after endoscopic resection, shown against a millimeter scale.

Given the neurological deficit and failure of conservative management, transforaminal endoscopic discectomy was indicated. The procedure was performed under general anesthesia with multimodal IONM, including transcranial motor-evoked potentials, somatosensory-evoked potentials, and free-run electromyography of multiple lower extremity muscle groups. Pre-decompression recordings demonstrated significantly reduced amplitudes and morphological alterations in left-sided motor-evoked potentials, predominantly in L4-innervated musculature, with stable contralateral signals (Fig. 2A).

Following fluoroscopic confirmation of the target level, discography was performed at L4-L5 with a total of 15 mL of solution composed of 2 mL of 1% methylene blue (10 mg/mL) and 13 mL of nonionic iodinated contrast medium (Omnipaque 300), giving a final methylene blue concentration of approximately 1.3 mg/mL (Fig. 2B). The herniation was classified as Lee zone 1 [12] (Fig. 2C). Transforaminal endoscopic access was established and directed toward the cranially migrated foraminal fragment. Methylene blue staining rendered 2 irregular extruded disc fragments clearly identifiable against the surrounding epidural tissue, enabling targeted resection while preserving adjacent neural structures (Fig. 2D). Together, the fragments measured 4.0 × 1.2 × 0.5 cm. Progressive improvement in neurophysiological signal quality was observed during decompression, with partial recovery of amplitude and morphology in the previously compromised potentials, without new deterioration events. Total operative time was 96 minutes and estimated blood loss was 10 mL. A total of 10 L of normal saline was used for continuous irrigation throughout the uniportal full-endoscopic procedure.

The patient was discharged on the day of surgery with minimal pain and has remained under regular outpatient follow-up. Functional outcomes improved substantially, with the ODI decreasing from baseline to 32.5% at 1 week postoperatively and to 10% at 1 month, accompanied by near-complete resolution of radicular symptoms. The visual analog scale decreased from 10 preoperatively to less than 1 at 1 month. At the 8-month follow-up, the ODI remained stable at 10%, reflecting sustained clinical improvement. The patient reported no significant pain and no longer required analgesic medication.

## Discussion

The clinical trajectory of this case illustrates the dynamic behavior of foraminal lumbar disc herniations. Abrupt neurological deterioration superimposed on a background of non-specific degenerative findings underscores the importance of prompt clinical and radiological reassessment when radicular symptoms worsen suddenly [13]. A notable strength of this report is the prospective documentation of IONM data at defined intraoperative time points, which provided an objective, quantifiable correlate of neural decompression rather than relying solely on subjective intraoperative or postoperative assessment.

In this anatomical location, the main imaging differential diagnoses include a nerve sheath tumor, a synovial cyst, and a perineural (Tarlov) cyst. A sequestered fragment that has lost continuity with the parent disc also enters the differential, and it is precisely that continuity which separates the 2 entities. In the present case the lesion showed signal characteristics consistent with disc material and, most importantly, clear continuity with the adjacent intervertebral disc. These features favored a migrated extruded fragment and made the alternative diagnoses unlikely.

Transforaminal endoscopic discectomy has demonstrated favorable outcomes for foraminal herniations, yet cranially migrated variants remain technically demanding [3,5]. The narrow working corridor limits instrument maneuverability and direct visual inspection of the foramen, and incomplete fragment resection is a recognized cause of clinical failure in this anatomy [4,5]. The use of a visual marker to distinguish disc material from surrounding structures is a logical response to this limitation.

Intradiscal dye injection for endoscopic guidance has evolved from the foundational transforaminal technique, in which discography with dye injection was integral to initial access and disc localization [6]. Selective chromatic identification was subsequently formalized by Kim et al. using indigo carmine [7], and methylene blue has since been applied in transforaminal endoscopic procedures, with Pirabakaran et al. reporting reductions in operative time and more complete fragment retrieval in a dedicated cohort [9]. The present case extends this application to a foraminal herniation with cranial migration, a subtype in which the working axis runs nearly parallel to the displaced fragment, and conventional fluoroscopic guidance provides limited spatial discrimination between the extruded material and adjacent soft tissue. In this geometry, the chromatic contrast offered by methylene blue may be proportionally more valuable than in standard central herniations.

Several technical considerations specific to methylene blue warrant discussion. Dye diffusion through the annular fissure before endoscopic access is established represents the principal limitation. Once annular integrity is compromised by extrusion, methylene blue may migrate into the epidural space, potentially staining structures beyond the target fragment [9]. The interval between injection and endoscopic dissection is therefore relevant, as prolonged delays may result in dye washout or non-selective diffusion. Concentration also matters, given that lower concentrations reduce the theoretical risk of neural irritation but yield weaker chromatic contrast [14]. In the in vitro comparison cited above, significant staining of bovine coccygeal discs occurred at 0.25 mg/mL and measurable cytotoxicity at 0.5 mg/mL. Those experiments involved sustained exposure of isolated disc cells rather than brief intradiscal exposure followed by continuous saline irrigation, so their thresholds are not directly transferable to the clinical setting. The final concentration used here, approximately 1.3 mg/mL, therefore exceeds the in vitro cytotoxicity threshold. Staining nonetheless appeared to remain localized to disc material without diffusing to adjacent neural elements, and no clinical or neurophysiological deterioration was observed. Even so, the narrow margin between effective staining and measurable toxicity supports the use of the lowest concentration that yields usable contrast. It should be noted that methylene blue stains both degenerated and normal nucleus pulposus, so the technique discriminates disc tissue from non-disc tissue rather than selectively identifying pathological material. This distinction is the mechanistically relevant one for fragment identification in this context.

A further element of this report is the simultaneous integration of IONM. The progressive recovery of motor-evoked potential amplitudes during fragment resection provided real-time functional confirmation that decompression was neurophysiologically effective, a feedback loop that dye staining alone cannot offer. While IONM has been described as a safety tool in endoscopic lumbar surgery [15], its combination with dye-guided anatomical targeting has rarely been described, and to our knowledge has not been reported in a cranially migrated foraminal herniation. The convergence of both strategies allowed a confident, directed resection rather than an exhaustive exploratory approach.

As a single case without a comparator group and with short-term follow-up, no causal inference can be drawn regarding either technique's independent contribution to the outcome. The separate effects of methylene blue staining and IONM cannot be disentangled. Recurrence cannot be excluded at longer follow-up. These limitations reinforce the need for prospective comparative series with standardized outcome measures.

## Conclusion

In this patient, methylene blue discography stained the extruded disc fragments and appeared to facilitate their identification during transforaminal endoscopic discectomy for a cranially migrated L4-L5 foraminal herniation, supporting a targeted resection in an anatomically constrained corridor. Concurrent IONM added functional confirmation of adequate neural decompression, addressing complementary aspects of surgical uncertainty that conventional fluoroscopy and endoscopic visualization alone may not resolve. Whether this combination reduces residual fragment rates or improves clinical outcomes in this specific herniation subtype cannot be determined from a single case, and prospective comparative studies with standardized outcome measures are needed.

## Author contributions

Conceptualization: Marcus Vinicius Brito Marques, Marcelo Botelho Soares de Brito; Methodology: Marcus Vinicius Brito Marques, Fabrício Guedes Machado; Formal analysis and investigation: Marcus Vinicius Brito Marques, Jorge Luis Marques Ponte; Visualization: Roney Souza de Sant’Anna; Writing - original draft preparation: Marcus Vinicius Brito Marques, Fabrício Guedes Machado; Writing - review and editing Marcelo Botelho Soares de Brito, Jorge Luis Marques Ponte, Roney Souza de Sant’Anna.

## Declaration of generative AI and AI-assisted technologies in the manuscript preparation process

During the preparation of this work, the author(s) used ChatGPT (OpenAI) for translation and textual improvement of the manuscript. The author(s) carefully reviewed and edited all AI-generated outputs as necessary and take full responsibility for the content of the published article.

## Ethical approval

This case report was also reviewed and approved by the Research Ethics Committee (CEP) of Mater Dei Hospital S.A. (Certificate of Presentation for Ethical Review [CAAE]: 96089226.8.0000.5128), in accordance with applicable local legislation and the principles of the Declaration of Helsinki.

## Patient consent

Written informed consent for publication of this case report, including the associated clinical details and imaging, was obtained from the patient.
